# Development and validation of a risk prediction model for postpartum urinary incontinence in primiparas with singleton pregnancies: a multicenter clinical investigation

**DOI:** 10.3389/fmed.2024.1453029

**Published:** 2024-11-06

**Authors:** Xiaofeng Huang, Huangna Qin, Lin Kong, Hongwei Xia, Lixiang Lan, Junqing Long

**Affiliations:** Department of Obstetrics, Maternal and Child Health Hospital of Guangxi Zhuang Autonomous Region, Nanning, Guangxi, China

**Keywords:** urinary incontinence, primiparas, risk factor, prediction nomogram, singleton pregnancy

## Abstract

**Background:**

Postpartum urinary incontinence (UI) is a serious condition that significantly affects the quality of life. Several studies have demonstrated that it is associated with pelvic floor dysfunction. This study aimed to develop and validate a UI risk prediction model to identify primiparas with singleton pregnancies at high risk.

**Methods:**

A multistage stratified random sampling process was used. UI was measured using the International Standard Consultation on Incontinence Questionnaire Form (a modified Bristol questionnaire, ICIQ-FLUTS). Records of 1,340 primiparas with singleton pregnancies were reviewed, and data were collected from January 2014 to December 2014 in multiple centers. A univariate logistic regression analysis was performed, followed by a multivariable logistic regression analysis of the data. Using bootstrap resampling, we constructed a nomogram to assess postpartum UI risk.

**Results:**

A total of 1,340 patients were enrolled, including 345 with postpartum UI and 995 with non-postpartum UI. The occurrence of postpartum UI was significantly related to the mode of delivery, family history of UI, coffee or tea consumption, antenatal UI, and frequent cough. The nomogram exhibited good discriminatory ability with a C-index of 0.718 (95% confidence interval: 0.684–0.752) and a bootstrap-corrected C-index of 0.716. Additionally, the calibration curve demonstrated that the predicted outcomes aligned well with the actual observations. Ultimately, the decision curve analysis indicated that the nomogram exhibited favorable clinical applicability.

**Conclusion:**

The decision curve analysis suggests that the nomogram could provide clinical value. The clinician will then feel more confident about making clinical recommendations regarding postpartum UI screening for primiparous women with singleton pregnancies.

## Introduction

Urinary incontinence (UI) is an involuntary leakage of urine that occurs unintentionally. UI is a serious condition that has a negative impact on quality of life and significantly affects society and families (1). After childbirth, UI is among the most common symptoms associated with pelvic floor dysfunction in women. Some studies have reported that UI affects 55% of women who give birth for the first time within 12 months postpartum (2). Therefore, this issue has received considerable research attention. Numerous studies have attempted to determine its pathogenesis and cause. A 4-year longitudinal study among 24,985 adult women revealed an overall UI incidence rate of 21.2 episodes per 1,000 person-years in females in China. The main risk factors include delivery (spontaneous and instrumental vaginal deliveries), cigarette smoking, chronic cough, diabetes, high body mass index (BMI), and older age (3). However, it is unclear how postpartum UI can be accurately predicted in advance.

As statistical analysis has evolved, an increasing number of medical prediction and intervention models are being utilized in clinical medicine (4). The predicted indicators suggest that we can intervene earlier in patients, potentially leading to greater benefits from the intervention. Multiple-factor models often yield better results than single-factor models in terms of diagnosis and prognosis prediction (5). Nomograms represent a visualization of a statistical model for individual prediction (6). Moreover, a nomogram has a relatively short learning curve and is relatively easy to use in a clinical setting. Consequently, it is necessary to develop a nomogram to predict the occurrence of postpartum UI in first-time pregnant women.

In this study, a multistage stratified random sampling process was applied. UI was measured using the International Consultation on Incontinence Questionnaire Form (a modified version of the Bristol questionnaire, ICIQ-FLUTS). Demographic and clinical characteristics were collected and analyzed. We developed a nomogram prediction model to examine the risk factors, predict interventions in advance, and prevent the occurrence of postpartum UI in first-time mothers. This may be used for diagnosing and treating pregnant women at high risk of UI based on scientific evidence. Clinicians will then feel more confident about making clinical recommendations regarding postpartum UI screening for primiparous women with singleton pregnancies.

## Methods

### Patients

Stratified random sampling was performed in multiple stages. Samples from the patients were obtained from maternal and child health hospitals in four cities (Nanning, Guilin, Liuzhou, and Pingguo) and two counties (Longsheng and Hepu) in the Guangxi Zhuang Autonomous Region between January 2014 and December 2014. The age of the pregnant women ranged from 18 to 46 years, with an average age of 35. The inclusion criteria were as follows: (1) The choice of samples was between cesarean section or vaginal delivery when the baby was a breech. (2) Complete clinical records and follow-up data were available for all the patients. (3) The participants did not perform pelvic floor muscle exercises. (4) Patients were first-time mothers. (5) Those who could participate in the questionnaire within 12 weeks of delivery. The exclusion criteria were as follows: (1) Patients with a critical surgical illness or severe internal disease; (2) patients with history of UI or urethral surgery. After screening for study inclusion and exclusion criteria, 1,340 cases were included in the study.

### Data collection

To assess postpartum incontinence status, a structured questionnaire was administered face-to-face, incorporating the International Consultation on Incontinence Questionnaire Form (a modified Bristol questionnaire, ICIQ-FLUTS) (7, 8). We then developed and added corresponding survey items. Factors including age, ethnic group, educational level, occupation, income, coffee or tea consumption, and family history of UI can influence the condition. Antenatal UI, postpartum UI, diabetes mellitus (DM), hypertension (HTN), frequent cough, frequent constipation, BMI, number of pregnancies, mode of delivery, epidural labor analgesia, perineal laceration during delivery, the bulge of the anterior and posterior vaginal wall, uterine prolapse, and breastfeeding were also included in the questionnaire. Participants were divided into postpartum UI (*n* = 345) and non-postpartum UI (*n* = 995) groups.

### Statistical analysis

The statistical analyses were performed using the Statistical Package for the Social Sciences software (version 26.0; IBM Corp, Armonk, NY, USA), and graphics were produced using R software [rms (9) in R version 3.6.2]. Categorical data are presented as numbers and proportions. We compared the groups using the chi-squared test. The predictors of postpartum UI were determined by univariate and multivariate logistic regression analysis UI. Based on the results of the multivariable logistic regression, a clinical prediction nomogram was developed using R software to evaluate the risk of postpartum UI. Calibration curves were used to determine whether the nomogram was calibrated. Decision curve analysis was applied to evaluate the clinical effectiveness of the prediction model. All tests were two-sided, and *p* < 0.05 was considered statistically significant.

## Results

### Comparison of postpartum and non-postpartum UI

Table 1 reveals a comparison of the outcomes between the two groups. The mode of delivery, family history of UI, drinking habits, antenatal UI, frequent cough, frequent constipation, and perineal laceration during delivery were factors that exhibited statistically significant differences between the groups. The postpartum UI group exhibited a higher proportion of these factors compared to the non-postpartum UI group. Other factors did not reveal statistically significant differences between the two groups. These results suggest that primiparas with singleton pregnancies and a family history of UI, consumption of coffee or tea, antenatal UI, frequent coughing, frequent constipation, and perineal lacerations during vaginal delivery are more likely to develop postpartum UI.

**Table 1:** Clinical characteristics of patients.

Variable	Postpartum UI group (*n* = 345)	Non-postpartum UI group (*n* = 995)	*p*-value
Mode of delivery, N (%)			0.001
Vaginal delivery	251 (72.8)	625 (62.8)	
Cesarean delivery	94 (27.2)	370 (37.2)	
Educational level, N (%)			0.369
College	133 (38.6)	362 (36.4)	
Secondary vocational school	87 (25.2)	284 (28.5)	
Junior high school	108 (31.3)	316 (31.8)	
Primary school	17 (4.9)	33 (3.3)	
Career, N (%)			0.093
Government functionary	13 (6.8)	55 (5.5)	
Worker	23 (6.7)	57 (5.7)	
Farmer	68 (19.7)	189 (19.0)	
Freelance work	97 (28.1)	316 (31.8)	
Business	37 (10.7)	98 (9.8)	
Teacher	20 (5.8)	56 (5.6)	
Salesperson	30 (8.7)	44 (4.4)	
Nurse	2 (0.6)	15 (1.5)	
Other	55 (15.9)	165 (16.6)	
Income, N (%)			0.968
<2,000¥	152 (44.1)	451 (45.3)	
2,000–5,000¥	148 (42.9)	421 (42.3)	
5,000–10,000¥	29 (8.4)	77 (7.7)	
>10,000¥	16 (4.6)	46 (4.6)	
BMI, N (%)			0.467
<18.5	21 (6.1)	80 (8.0)	
18.5–24.9	277 (80.3)	775 (77.9)	
≥25.0	47 (13.6)	140 (14.1)	
Family history of UI, yes, N (%)	16 (4.6)	4 (0.5)	<0.001
Drinking coffee or tea, yes, N (%)	51 (14.8)	92 (9.2)	0.004
Smoking history, yes, N (%)	7 (2.0)	12 (1.2)	0.265
Antenatal UI, yes, N (%)	111 (32.2)	18 (1.8)	<0.001
Hypertension (HTN), yes, N (%)	3 (0.9)	10 (1.0)	0.825
Diabetes mellitus (DM), yes, N (%)	14 (4.1)	31 (3.1)	0.402
Frequent cough, yes, N (%)	14 (4.1)	11 (1.1)	<0.001
Frequent constipation, yes, N (%)	126 (36.5)	278 (27.9)	0.003
Number of pregnancies, N (%)			0.754
1 time	182 (52.8)	547 (55.0)	
2 times	98 (28.4)	274 (27.5)	
≥3 times	65 (18.8)	174 (17.5)	
Delivery perineal lacerations, yes, N (%)	175 (50.7)	396 (39.8)	<0.001
Epidural labor analgesia, yes, N (%)	162 (47.0)	424 (42.6)	0.161
Breastfeeding, yes, N (%)	316 (91.6)	930 (93.5)	0.24
Bulge of anterior vaginal wall, yes, N (%)	136 (39.4)	335 (33.7)	0.054
Bulge of posterior vaginal wall, yes, N (%)	32 (9.3)	107 (10.8)	0.438
Uterine prolapse, yes, N (%)	28 (8.1)	82 (8.2)	0.942

### Selected predictors for model

Univariate logistic multivariable regression analysis was used in the postpartum UI group. The results are demonstrated in Table 2. Variables with statistically significant differences, including mode of delivery (*p* = 0.001), family history of UI (*p* < 0.001), drinking habits (*p* = 0.004), antenatal UI (*p* < 0.001), frequent cough (*p* = 0.001), frequent constipation (*p* = 0.003), and perineal laceration (*p* < 0.001) were included in the univariate logistic multivariable regression analysis. Stepwise backward logistic regression was used to analyze multivariate data. The results exhibited that the occurrence of postpartum UI was significantly related to the mode of delivery (*p* = 0.002), family history of UI (*p* < 0.001), coffee or tea consumption (*p* = 0.031), antenatal UI (*p* < 0.001), and frequent cough (*p* < 0.001).

**Table 2:** Univariate logistic multivariable regression analysis of postpartum UI.

Variable	*β*	Odds ratio (95% CI)	*p*-value
Family history of UI	2.265	9.629 (3.500–26.488)	<0.001
Antenatal UI	3.248	25.747 (15.337–43.223)	<0.001
Delivery perineal lacerations	0.443	1.557 (1.217–1.992)	<0.001
Uterine prolapse	−0.017	0.983 (0.629–1.539)	0.942
Educational level	−0.007	0.993 (0.868–1.135)	0.916
Hypertension	−0.146	0.864 (0.236–3.158)	0.825
Income	−0.031	0.970 (0.833–1.130)	0.694
BMI	0.071	0.931 (0.713–1.215)	0.6
Career	−0.018	0.982 (0.932–1.035)	0.499
Number of pregnancies	−0.06	0.942 (0.804–1.103)	0.456
Bulge of posterior vaginal wall	−0.164	0.848 (0.560–1.285)	0.438
Diabetes mellitus	0.274	1.315 (0.691–2.503)	0.404
Smoking history	0.529	1.696 (0.662–4.344)	0.271
Breastfeeding	−0.272	0.762 (0.483–1.201)	0.242
Epidural labor analgesia	0.176	1.192 (0.932–1.525)	0.161
Bulge of anterior vaginal wall	0.248	1.282 (0.996–1.651)	0.054
Drinking coffee or tea	0.532	1.703 (1.180–2.457)	0.004
Frequent constipation	0.395	1.484 (1.145–1.923)	0.003
Mode of delivery	0.458	1.581 (0.207–2.070)	0.001
Frequent cough	1.331	3.784 (1.701–8.416)	0.001

Based on the above results, postpartum and non-postpartum UI groups were compared. Five factors (vaginal delivery, having a family history of UI, drinking coffee or tea, experiencing antenatal UI, and having a frequent cough) increase the risk of postpartum UI in primiparas with singleton pregnancies.

### Predictive nomogram for the risk of postpartum UI

**Figure 1:**
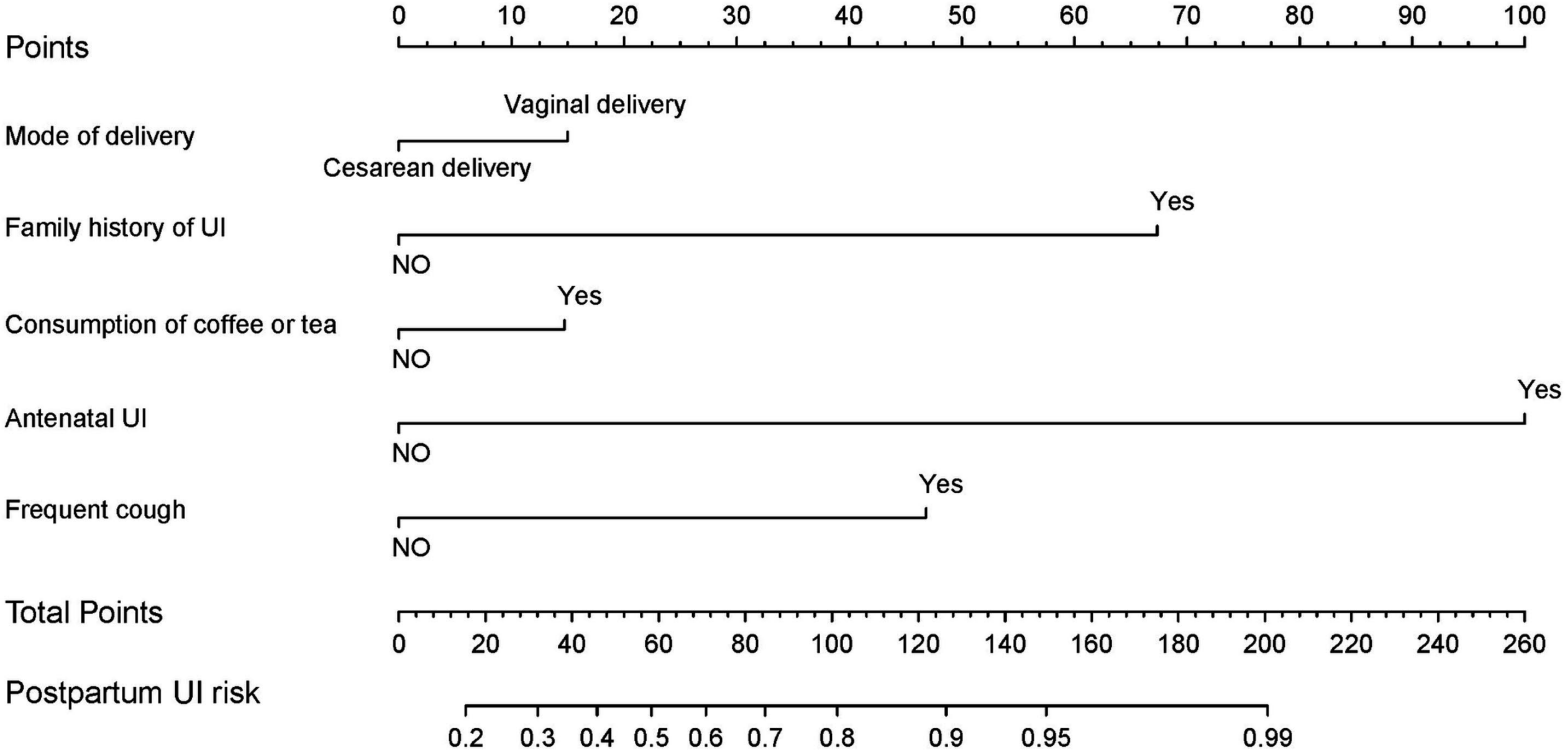
Nomogram developed with the mode of delivery, family history of UI, drinking coffee or tea, antenatal UI, and frequent cough.

Based on the analysis of multivariate logistic regression, the *p*-value was provided. Five significant predictors were considered when establishing a nomogram for primiparas of singleton pregnancy with postpartum UI prediction (Figure 1). Based on the sum of these five parameters (mode of delivery, family history of UI, consumption of coffee or tea, antenatal UI, and frequent cough), a total score was generated. Briefly, we utilized the nomogram by placing the patient’s characteristics on each axis. A straight line was drawn up to the point axis to determine the points assigned to each variable. All points for the variables were summed, and the probability of experiencing postpartum UI was calculated by drawing a line from the total points axis to the bottom line (Table 3).

**Table 3:** Multivariate logistic multivariable regression analysis of postpartum UI.

Variable	*β*	Odds ratio (95% CI)	*p*-value
Family history of UI	2.154	8.623 (2.834–26.235)	<0.001
Antenatal UI	3.207	24.693 (14.608–41.739)	<0.001
Frequent constipation	0.282	1.325 (0.982–1.788)	0.066
Drinking coffee or tea	0.469	1.598 (1.043–2.448)	0.031
Frequent cough	1.573	4.820 (2.013–11.542)	<0.001
Mode of delivery	0.486	1.626 (1.196–2.212)	0.002
Delivery perineal lacerations	0.194	1.214 (0.858–1.719)	0.273

### Performance of the nomogram

**Figure 2:**
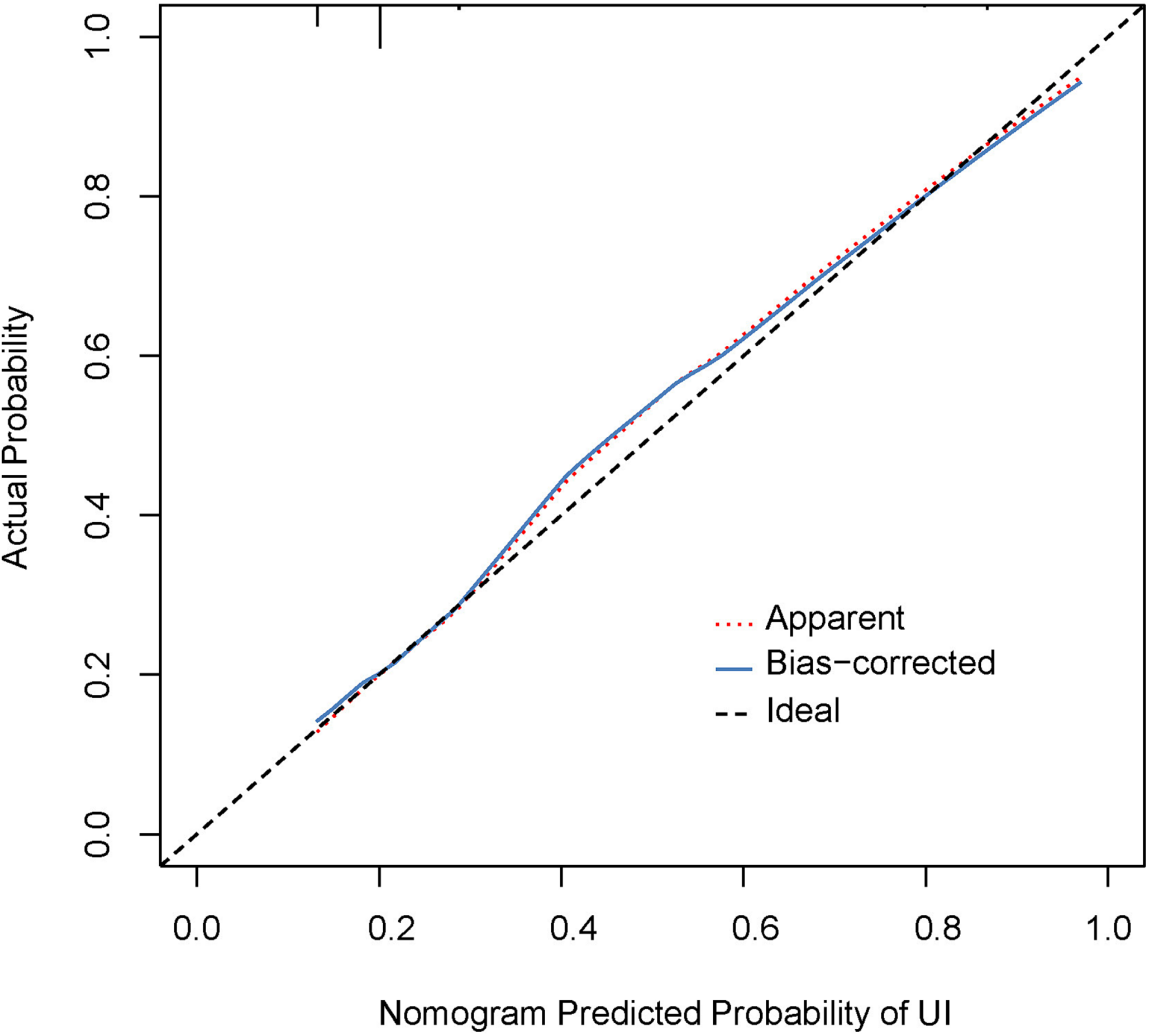
Calibration curves for the nomogram. The red dotted line indicates the performance of the entire cohort (*n* = 1,340), whereas the black solid line indicates bias correction using bootstrapping (*B* = 1,000 repetitions) and represents the observed performance from the nomogram.

As part of the internal validation, we employed 1,000 repetitions of bootstrapping to assess the performance of the nomogram. A C-index of 0.718 (95% confidence interval [CI]: 0.684–0.752) was obtained, suggesting that the nomogram was an accurate predictor of primiparas of singleton pregnancy with postpartum UI. It also had a relatively high bootstrap-corrected C-index of 0.716. The prediction model exhibited a favorable discrimination performance. Using a calibration curve, we assessed the consistency of the occurrence of postpartum UI between the predicted probability of the nomogram and the actual observed probability (Figure 2). Overall, the nomogram performed satisfactorily.

### Clinical use of nomogram

**Figure 3:**
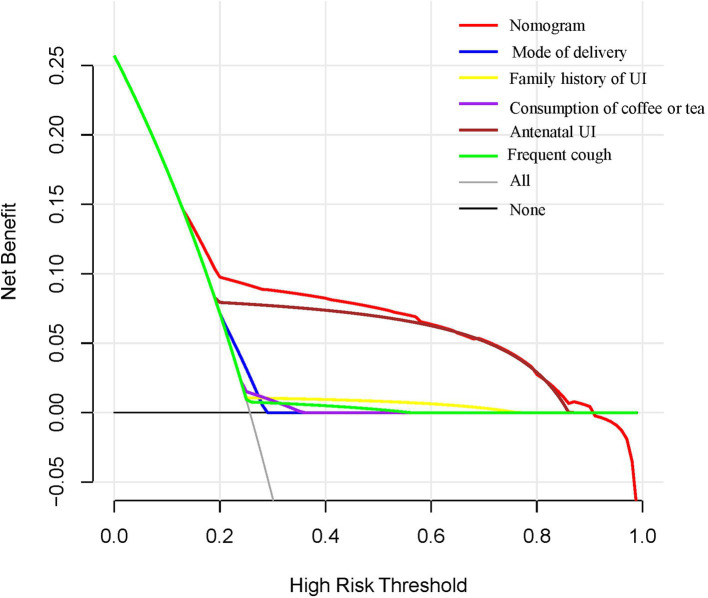
DCA for the nomogram, mode of delivery, family history of UI, drinking coffee or tea, antenatal UI, and frequent cough. The *x*- and *y*-axes reflect threshold probability and net benefit, respectively. The red line represents the nomogram.

A decision curve analysis (DCA) was then used to evaluate the clinical usefulness of the nomogram. The DCA model with a single predictor is presented in Figure 3. Based on the DCA curves, using the nomogram for screening strategies was predicted to provide more net benefit than screen-none or screen-all schemes when the threshold probability for patients or clinicians was between 15 and 59%. In this range, a nomogram is more predictive than a single predictor.

## Discussion

Prior studies have noted that people with UI often suffer from a devastated quality of life because of this condition (10). As a result of women’s lack of attention and lack of timely treatment, it leads to low outpatient physician visit rates. The initial objective of this study was to identify risk factors for postpartum UI in primiparas.

According to this study, the results indicate that family history of UI, alcohol consumption, antenatal UI, frequent coughing, frequent constipation, and perineal laceration are risk factors for postpartum UI in primiparas. Additionally, the multivariate analysis revealed that vaginal delivery, family history of UI, alcohol consumption, antenatal UI, and frequent coughing were independent risk factors for postpartum UI.

According to other reports, vaginal delivery has been associated with neuromuscular damage to the pelvic floor well before the emergence of clinically significant symptoms of pelvic floor dysfunction (11). Injury to the ligaments and pelvic floor muscles can result in a weak pelvic floor, causing mobility in the bladder, neck, and urethra, which can lead to urethral sphincter incompetence (12). We surveyed 1,340 primiparous women. The total number of UI cases was 345 (25.8%), of which 28 (58.4%) were mixed UI cases. Postpartum UI in primiparas occurred more frequently after vaginal delivery than cesarean delivery (28.7% versus 20.3%). Multivariate analysis revealed that vaginal delivery was an independent risk factor for UI (odds ratio [OR] 1.626, 95% CI: 1.196–2.212). It may be linked to fewer pelvic floor injuries in cesarean than in vaginal deliveries.

Moreover, a family history of UI, alcohol consumption, antenatal UI, and frequent cough are independent risk factors for UI. This mechanism may be related to the lack of connective tissue in individuals with a positive family history. Additionally, some pregnant women with a family history of UI exhibit abnormal expression of ZFP521, ADAMTS16, and CIT gene proteins. This leads to weakened binding of the CIT gene to the cerebral cortex striatum, resulting in dysfunction of the cerebral cortex and subcortical centers, leading to involuntary urination (13, 14). Chronic cough can cause increased abdominal pressure, indirectly leading to increased bladder pressure (15). When the dynamic urine pressure exceeds the pressure on the unit area of the urethral sphincter, the urethral sphincter opens, leading to uncontrolled urine overflow (16, 17). According to the report, 84.5% (*n* = 279) of pregnant women had experienced UI, and coffee consumption was identified as a major risk factor for UI (18). The habit of drinking tea has been identified as a risk factor associated with an increased prevalence of UI (19). The reason may be that excessive consumption of these beverages (coffee and tea) was associated with an OR of 2.98 (95% CI: 1.26–7.03) for overactive bladder (20). Currently, there is only one operational and implemented mode for postpartum UI that is associated with the delivery method (21). Although some risk factors for postpartum UI were mentioned in the literature (22, 23), a comprehensive prediction model has not been proposed for use in clinical contexts. Therefore, a straightforward and practical model is still lacking. A nomogram was plotted to facilitate its clinical application. A screening nomogram of quantitative indicators has been constructed based on factors such as vaginal delivery, family history of UI, consumption of coffee or tea, antenatal UI, and frequent cough. This is a particularly encouraging result, as the model demonstrates excellent predictive and discrimination abilities. DCA curves exhibit that using the nomogram for screening strategies is predicted to provide more net benefit than screen-none or screen-all schemes when the threshold probability for patients or clinicians is between 15 and 59%. These findings also demonstrate the value of this model in identifying high-risk primiparas. Drinking interventions combined with pelvic floor rehabilitation for the treatment of postpartum pelvic floor function can help prevent UI in primiparas. This study has some limitations, such as the small number of postpartum primiparas with UI included. Further studies are required to increase the sample size of this study. Our findings require additional clinical validation for practical application.

The discovery that vaginal delivery exacerbates UI has important significance for clinical practice and research. In clinical practice, this finding highlights the importance of early identification and intervention, and healthcare providers can strengthen the support and management of pregnant women through screening and education to reduce the discomfort and psychological burden of UI. Based on this finding, it is particularly important to develop individualized postnatal rehabilitation programs. At the research level, this finding adds empirical support to the existing literature by highlighting the relationship between delivery mode and UI. This provides a direction for further exploration of prevention and treatment strategies and prompts investigators to focus on other potential factors affecting incontinence. Future research should continue to explore the long-term effects of different delivery methods on pelvic floor function and develop targeted rehabilitation and prevention programs that ultimately improve women’s quality of life. There are many treatments for female incontinence, including surgical and medical interventions, and studies have demonstrated that ospemifene is an effective potential therapy (24, 25). Accordingly, for some patients, rehabilitation therapy and medication cannot be improved, and active surgical measures should be taken to improve the quality of life of patients.

Retrospective questionnaire studies have some limitations when exploring postpartum UI in pregnant women. First, retrospective studies rely on participants memories and self-reports, which can lead to information bias. Second, retrospective studies often fail to control for all potential confounding factors, which may affect the judgment of causality. Moreover, retrospective surveys may face selection bias in sample selection and may fail to represent a broader population. As a result, to obtain more comprehensive and accurate results, we will combine prospective studies and multiple data-collection methods in the future. However, as a regional quality control center, our hospital has a significant advantage in the retrospective investigation of postpartum UI in pregnant women. First, there is a wealth of clinical data and case resources to ensure the representativeness and reliability of the sample. Second, through the support of regional quality control networks, we can promote multicenter cooperation and improve the breadth and impact of research. The findings of this study provide high-quality clinical evidence to encourage healthcare institutions in the region to value and improve patient care and treatment options.

## Conclusion

We have developed a practical nomogram to predict the probability of postpartum UI in primiparas. Clinicians will then feel more confident about making clinical recommendations regarding postpartum UI screening for primiparas. This study may assist clinicians in developing more tailored treatment recommendations for high-risk primiparas who may be at risk of developing postpartum UI.

## Data Availability

The raw data supporting the conclusions of this article will be made available by the authors, without undue reservation.
